# Pioglitazone abolishes cognition impairments as well as BDNF and neurotensin disturbances in a rat model of autism

**DOI:** 10.1242/bio.041327

**Published:** 2019-04-29

**Authors:** Thiago B. Kirsten, Renato C. Casarin, Maria M. Bernardi, Luciano F. Felicio

**Affiliations:** 1 Department of Pathology, School of Veterinary Medicine, University of São Paulo, São Paulo 05508-270, Brazil; 2 Environmental and Experimental Pathology, Paulista University, São Paulo 04026-002, Brazil; 3 Graduate Program of Dentistry, Paulista University, São Paulo 04026-002, Brazil

**Keywords:** Prenatal, Maternal immune activation, T-maze spontaneous alternations test, LPS, Neuropeptides

## Abstract

We have shown that exposure of rats to lipopolysaccharide (LPS) during gestation induces autistic-like behaviors in juvenile offspring and pioglitazone post treatment corrects social and communication deficits. The first objective of the present study was to evaluate the cognition of the rats, because this is also a behavioral sphere committed in autism. Second, biomarkers related to pioglitazone pathways and autism were studied to try to understand their mechanisms. We used our rat model of autism and pioglitazone was administered daily to these young offspring. T-maze spontaneous alternations tests, plasma levels of brain-derived neurotrophic factor (BDNF), beta-endorphin, neurotensin, oxytocin, and substance P were all studied. Exposure of rats to LPS during gestation induced cognitive deficits in the young offspring, elevated BDNF levels and decreased neurotensin levels. Daily postnatal pioglitazone treatment abolished cognition impairments as well as BDNF and neurotensin disturbances. Together with our previous studies, we suggest pioglitazone as a candidate for the treatment of autism, because it improved the responses of the three most typical autistic-like behaviors. BDNF and neurotensin also appeared to be related to the autistic-like behaviors and should be considered for therapeutic purposes.

## Abstract (precis)

**Summary:** Exposure of rats to lipopolysaccharide during gestation induced autistic-like behaviors in the juvenile offspring. Daily postnatal pioglitazone treatment abolished cognition impairments as well as brain-derived neurotrophic factor and neurotensin disturbances.

## INTRODUCTION

Autism spectrum disorder (ASD) is a developmental disorder that is characterized by social, communicative and cognitive deficits and has a higher prevalence in males (DSM-IV, 1994). The incidence of autism is considered high in the population all over the world: about 1 in 59 children have been identified with ASD (Baio et al., 2018). Although it is considered that the etiology of autism is unknown, genetic and environmental factors (such as prenatal infections and maternal dietary disturbs) have been described as autistic triggers (Herbert, 2010; Johnson et al., 2013; Theoharides et al., 2009).

We have shown that exposure of rats to lipopolysaccharide (LPS, gram-negative bacteria endotoxin) during gestation [gestational day (GD) 9.5] induces socialization and communication deficits as well as repetitive/restricted behavior in juvenile offspring. These behavioral impairments are found just in males and not in the female offspring (Kirsten et al., 2010a, 2012; Kirsten and Bernardi, 2017). Therefore, our model of exposure of rats to LPS during gestation induces autistic-like behaviors in juvenile offspring (Kirsten et al., 2012). We have also shown peripheral interleukin (IL)-1 beta and IL-6 levels increase in adult and young male offspring, respectively (Kirsten et al., 2013, 2018); results that have been described in autistic patients (Li et al., 2009; Jyonouchi et al., 2001; Al-Ayadhi, 2005). Additionally, the mechanisms that are involved in fetal brain disturbances include the production and release of proinflammatory cytokines within the maternal environment (circulation and placenta) (Ashdown et al., 2006; Urakubo et al., 2001; Cai et al., 2000).

There is no effective treatment for ASD, and a small number of drugs are authorized by the US Food and Drug Administration (FDA) (McPheeters et al., 2011). These drugs have limited efficacy and are able to treat only some ASD symptoms and can trigger adverse effects (Wink et al., 2010). Therefore, the present study proposed to use our rat model of autism to find new pharmacological approaches for autism. Pioglitazone was selected as the postnatal treatment to inhibit or relieve the behavioral and immune disturbances induced after the exposure of rats to LPS during gestation. Pioglitazone belongs to the thiazolidinediones class, which act as peroxisome proliferator-activated receptor gamma (PPARγ) agonists (Gillies and Dunn, 2000; Berger and Wagner, 2002). Pioglitazone is an anti-diabetic drug that is authorized by the FDA to treat type 2 diabetes mellitus (insulin sensitizing effect) (Hanefeld and Belcher, 2001). Besides the anti-diabetic affect, thiazolidinediones has anti-inflammatory properties and has been suggested to treat inflammatory and neurological diseases such as inflammatory bowel disease, psoriasis, atherosclerosis, multiple sclerosis and Alzheimer's (Duval et al., 2002; Katayama et al., 2003; Bongartz et al., 2005; Feinstein, 2003; Landreth, 2006).

There are pilot studies showing that pioglitazone treatment associated with other pharmacological and behavioral therapies induce some clinical improvements in autistic patients. For example, daily pioglitazone treatment attenuated irritability, lethargy, stereotypy and hyperactivity in some autistic children, without significant side effects (Boris et al., 2007). Similarly, pioglitazone potentiated the risperidone effects in ASD patients (Ghaleiha et al., 2015).

Using our rat model of autism, we have demonstrated that pioglitazone treatment corrects social and communication deficits as well as elevated plasma IL-6 levels (Kirsten et al., 2018). The first objective of the present study was to evaluate the cognition of the rats, because this is also a behavioral sphere committed in autism. Second, biomarkers related to pioglitazone pathways and ASD were studied to try to understand their mechanisms.

## RESULTS

**Fig. 1.**
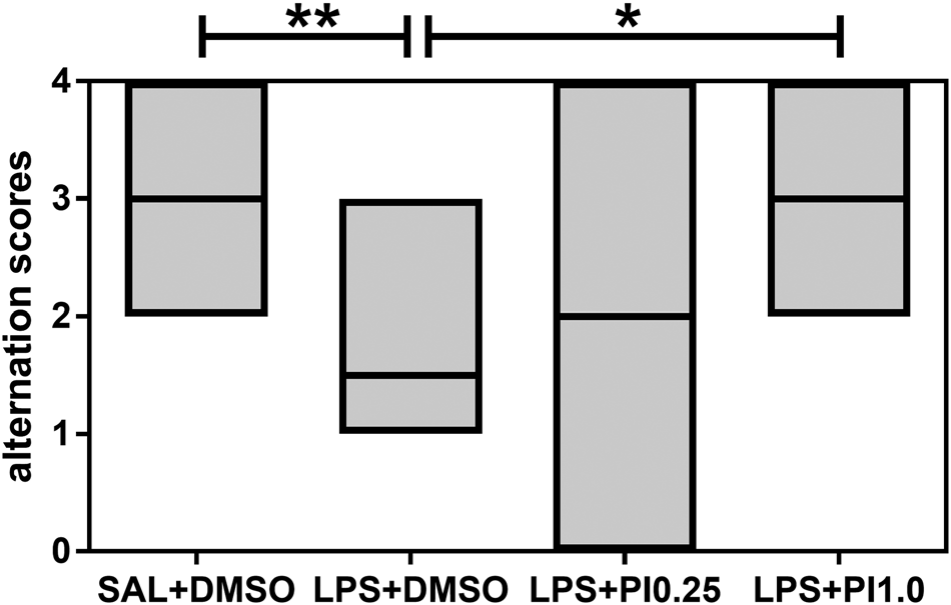
**T-maze.** The effects of prenatal LPS (100 µg/kg at GD 9.5) and postnatal pioglitazone [0.25 and 1.0 mg/kg/day between postnatal days (PND) 21 and 29] exposures on the T-maze spontaneous alternation test in juvenile male rat offspring. SAL+DMSO, prenatal saline injection and postnatal daily DMSO injection; LPS+DMSO, prenatal LPS injection and postnatal daily DMSO injection; LPS+PI0.25, prenatal LPS injection and postnatal pioglitazone 0.25 mg/kg/day; LPS+PI1.0, prenatal LPS injection and postnatal pioglitazone 1.0 mg/kg/day (*n*=8 rats/group). *P<0.05, **P<0.01 (Kruskal–Wallis test followed by Dunn's test). The data are expressed as the median (minimum and maximum).

Fig. 1 shows the effects of prenatal LPS and postnatal pioglitazone exposure on the T-maze spontaneous alternation test of the rats. The performance in the T-maze task was different between groups (KW=13.48, *P*=0.0037). Prenatal LPS exposure decreased T-maze spontaneous alternation in the offspring compared with the control group (*P*<0.01). Although postnatal pioglitazone treatment with 0.25 mg/kg/day did not affect the performance in the T-maze task, postnatal pioglitazone treatment with 1.0 mg/kg/day increased spontaneous alternation in the rats prenatally exposed to LPS (LPS+PI1.0 group versus LPS+DMSO group, *P*<0.05) to the same levels as those in the control group. Thus, prenatal LPS induced cognitive inflexibility, and postnatal pioglitazone treatment abolished these impairments.

**Fig. 2.**
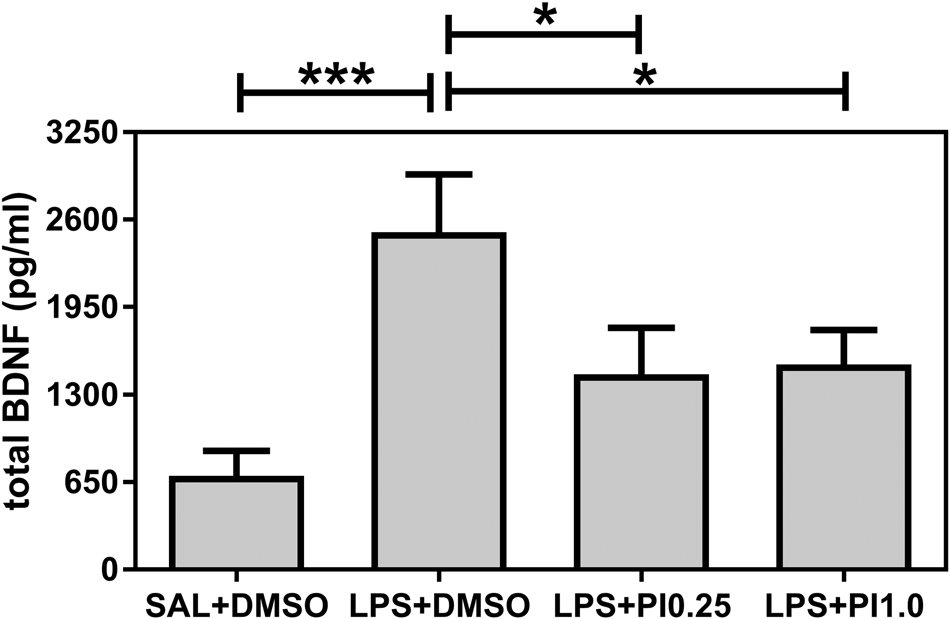
**BDNF.** The effects of prenatal LPS (100 µg/kg at GD 9.5) and postnatal pioglitazone (0.25 and 1.0 mg/kg/day between PND 21 and 29) exposures on the total BDNF plasma levels in juvenile male rat offspring. SAL+DMSO, prenatal saline injection and postnatal daily DMSO injection; LPS+DMSO, prenatal LPS injection and postnatal daily DMSO injection; LPS+PI0.25, prenatal LPS injection and postnatal pioglitazone 0.25 mg/kg/day; LPS+PI1.0, prenatal LPS injection and postnatal pioglitazone 1.0 mg/kg/day (*n*=8 rats/group). *P<0.05, **P<0.01, ***P<0.001 (one-way ANOVA followed by the Fisher's LSD test). The data are expressed as the mean±s.e.m.

Fig. 2 shows the effects of prenatal LPS and postnatal pioglitazone exposure on the total BDNF plasma levels of the rats. The BDNF levels were affected by the treatments [*F*(3/28)=5.43, *P*=0.0045]. Prenatal LPS exposure (LPS+DMSO group) increased the total BDNF levels compared with the control group (SAL+DMSO, *P*<0.001). Both doses (0.25 and 1.0 mg/kg/day) of the post-treatment with pioglitazone decreased BDNF levels in the rats that were prenatally exposed to LPS (versus LPS+DMSO group, *P*<0.05 for both doses) to the same levels as those in the control group.

**Fig. 3.**
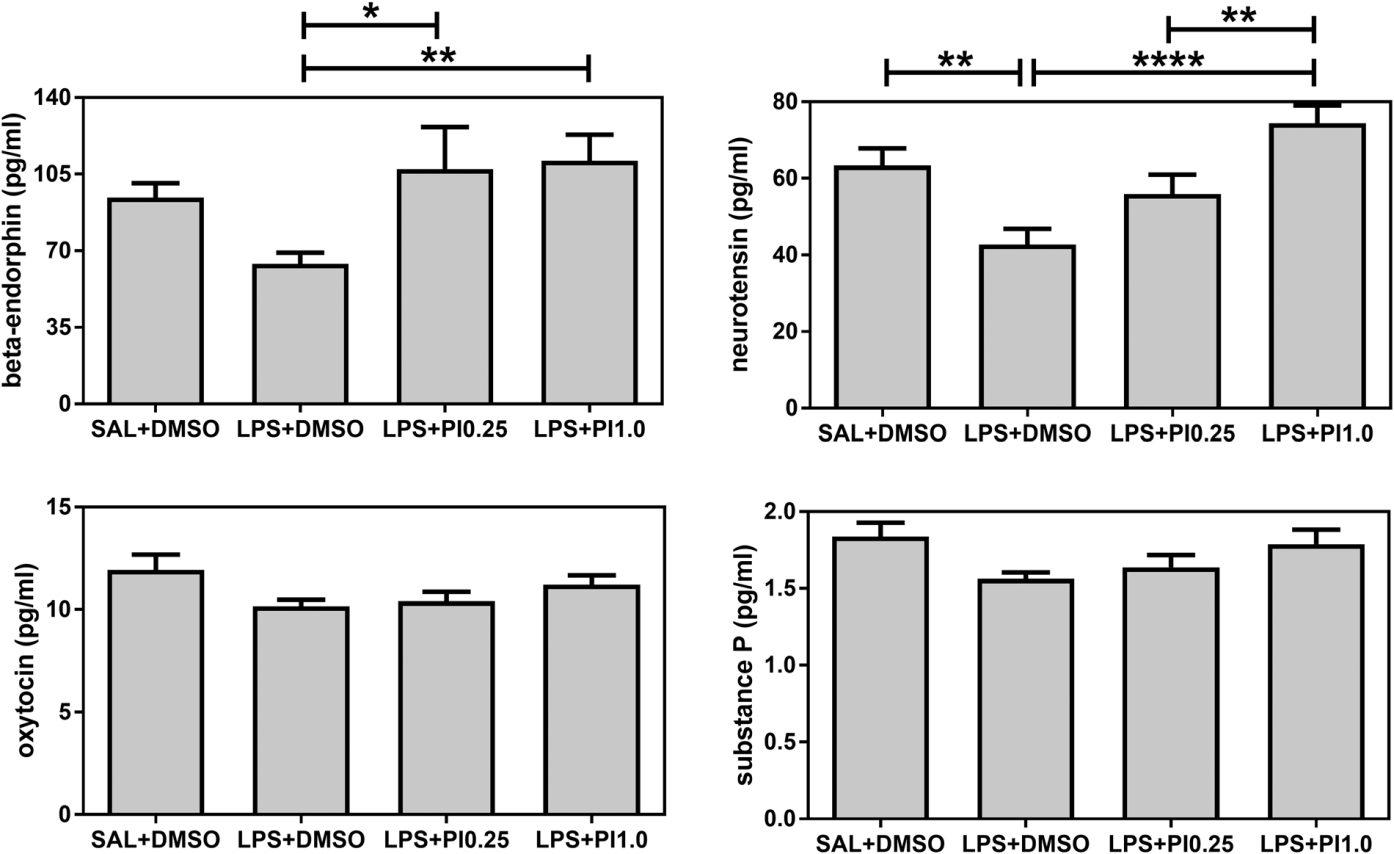
**Neuropeptides.** The effects of prenatal LPS (100 μg/kg at GD 9.5) and postnatal pioglitazone (0.25 and 1.0 mg/kg/day between PND 21 and 29) exposures on beta-endorphin, neurotensin, oxytocin and substance P plasma levels in juvenile male rat offspring. SAL+DMSO, prenatal saline injection and postnatal daily DMSO injection; LPS+DMSO, prenatal LPS injection and postnatal daily DMSO injection; LPS+PI0.25, prenatal LPS injection and postnatal pioglitazone 0.25 mg/kg/day; LPS+PI1.0, prenatal LPS injection and postnatal pioglitazone 1.0 mg/kg/day (*n*=8 rats/group). *P<0.05, **P<0.01, ***P<0.001, ****P<0.0001 (one-way ANOVA followed by the Fisher's LSD test). The data are expressed as the mean±s.e.m.

Fig. 3 shows the effects of prenatal LPS and postnatal pioglitazone exposure on the beta-endorphin, neurotensin, oxytocin and substance P plasma levels of the rats. Both beta-endorphin and neurotensin levels were affected by the treatments [*F*(3/28)=3.15, *P*=0.0407 and *F*(3/28)=8.41, *P*=0.0004, respectively], whereas oxytocin and substance P levels were not affected by the treatments [*F*(3/28)=2.34, *P*=0.0944 and *F*(3/28)=2.58, *P*=0.0735, respectively]. Prenatal LPS exposure (LPS+DMSO group) did not affect beta-endorphin levels compared with the control group (SAL+DMSO). Both pioglitazone doses (LPS+PI0.25 and LPS+PI1.0 groups) increased the beta-endorphin levels compared with the LPS group (versus LPS+DMSO, *P*<0.05 and *P*<0.01, respectively). Prenatal LPS exposure increased neurotensin levels compared with the control group (SAL+DMSO, *P*<0.01). Although postnatal pioglitazone treatment with 0.25 mg/kg/day did not affect the neurotensin levels compared with the LPS group it recovered the levels to the same levels as those in the control group. Postnatal pioglitazone treatment with 1.0 mg/kg/day increased the neurotensin levels in the rats prenatally exposed to LPS (LPS+PI1.0 group versus LPS+DMSO group, *P*<0.0001) to the same levels as those in the control group. Thus, prenatal LPS decreased neurotensin levels, and postnatal pioglitazone treatment recovered this disturbance.

## DISCUSSION

The most typical symptoms found in ASD are social, communicative and cognitive deficits, such as cognitive inﬂexibility and repetitive/restricted behavior (DSM-IV, 1994). We have shown that exposure of rats to LPS during gestation impairs communication in terms of ultrasonic vocalization (40 and 50 kHz), socialization (play behavior), induces repetitive/restricted behavior in terms of self-grooming and cognitive inﬂexibility (T-maze) in male offspring (Kirsten et al., 2010a, 2012, 2015a,b; Kirsten and Bernardi, 2017). Therefore, our model of exposure of rats to LPS during gestation induces autistic-like behaviors in juvenile offspring.

Considering no effective treatment yet exists for autism, we started to search for new treatments for autistic symptoms using our rat model. We selected pioglitazone because although it is originally an anti-diabetic drug, it also exerts anti-inflammatory effects in several cell types (Berger and Wagner, 2002; Hanefeld and Belcher, 2001). Moreover, there are pilot studies showing that pioglitazone treatment associated with other pharmacological and behavioral therapies induces some clinical improvements in autistic patients (Boris et al., 2007).

We have demonstrated that pioglitazone treatment corrects social and communication deficits (Kirsten et al., 2018). Our present results revealed that postnatal pioglitazone treatment with 1.0 mg/kg/day abolished the decreased T-maze spontaneous alternation induced by prenatal LPS. Together with our previous study (Kirsten et al., 2018), we suggest pioglitazone as a candidate for the treatment of autism, since it improved the responses of the three most typical autistic-like behaviors.

BDNF is a member of the neurotrophins family and is found in the central nervous system and peripheral blood. It is involved with morphological, developmental and neuronal processes, such as synaptogenesis and synaptic plasticity (Binder and Scharfman, 2004). Several autistic patients present elevated serum (Ricci et al., 2013), plasma (Correia et al., 2010), postmortem brain (Garcia et al., 2012) and neonatal cord blood (Nelson et al., 2001) levels of BDNF. Exposure of rats to LPS during gestation increased BDNF plasma levels. Thus, our rat model of autism also successfully reproduced the BDNF disturbance found in patients.

Both doses of postnatal pioglitazone treatment (0.25 and 1.0 mg/kg/day) abolished the BDNF disturbance induced by prenatal LPS. Although there are few studies about the relation between pioglitazone and BDNF, it is known that memory impairment, BDNF disturbance and oxidative damage induced by β-amyloid in an animal model of Alzheimer's disease are reversed by chronic administration of pioglitazone (Prakash and Kumar, 2014). Moreover, just like pioglitazone, BDNF has an anti-diabetic effect, ameliorating glucose metabolism and pancreatic dysfunction in type 2 diabetic mice (Yamanaka et al., 2007). Therefore, the BDNF disturbance induced by prenatal LPS was abolished by pioglitazone treatment.

Beta-endorphin, neurotensin, oxytocin and substance P plasma levels were studied because of their relationship with autism. There are reports of increased (Tordjman et al., 2009), decreased (Gillberg, 1992; Weizman et al., 1988) and unaltered (Angelidou et al., 2010) plasma/serum levels of beta-endorphin in autistic patients. Neurotensin levels have been found to be increased in the sera of 3-year old (Angelidou et al., 2010) and 6–12 year-old children with autism (Tsilioni et al., 2014). There are several studies reporting that ASD could be a result of oxytocin system disturbances early in life (Quattrocki and Friston, 2014). Oxytocin levels have been found to be decreased in the saliva of adolescent boys with autism (Bakker-Huvenaars et al., 2018), as well as in the plasma of Chinese autistic patients (Zhang et al., 2016). However, another group of autistic children and adolescents exhibited elevated serum oxytocin levels (Yang et al., 2017). Finally, substance P is also considered a possible factor in ASD because it is a product of the *Tachykinin 1* gene (*TAC1*), which is located in a candidate region for ASD (Marui et al., 2007). However, both serum (Angelidou et al., 2010) and neonatal blood (Nelson et al., 2001) of children with ASD present similar levels of substance P compared with control subject levels.

The apparent contradictory findings in the literature about the relationship between neuropeptides and autism may be explained by the presumably multifactorial etiopathogenesis of autism. Autism may be a result from a complex interaction between genetic and environmental factors (Posar and Visconti, 2017). Possible triggers include several chromosomal and gene disturbances, air pollutants, pesticides and other endocrine-disrupting chemicals, prenatal infections, mitochondrial dysfunction, electromagnetic pollution, diet modifications and possible epigenetic mechanisms (Posar and Visconti, 2017; Tordjman et al., 2014; Landrigan, 2010; Larsson et al., 2005).

Apparently, prenatal LPS exposure did not affect beta-endorphin, oxytocin and substance P plasma levels. Thus, the induction of autistic-like effects after the exposure of rats to LPS during gestation does not appear to be related to these neuropeptide pathways. However, prenatal LPS decreased neurotensin levels and postnatal pioglitazone treatment with 1.0 mg/kg/day abolished this disturbance.

There are a few studies about neurotensin and autism, and they report that autism increases neurotensin serum levels in children with autism (Angelidou et al., 2010; Tsilioni et al., 2014). We would expect that prenatal LPS should increase neurotensin levels because neurotensin may act like a neurotoxic factor and stimulating microglia, which activates the mammalian target of rapamycin (mTOR) signaling kinase and stimulates IL-1 beta gene expression (Patel et al., 2016; Ghanizadeh, 2010). Speaking of which, we have shown that prenatal LPS increases both striatal mTOR (Kirsten et al., 2015a) and serum IL-1 beta (Kirsten et al., 2013) levels in rats.

Neurotensin is widely distributed in the nervous system and peripherally, with neurotransmitter or neuromodulator roles (Vincent et al., 1999), including for cognition (Dobner, 2005). PD149163 administration, which is a neurotensin NTS1-receptor agonist, improves memory performance in Norway rats (Keiser et al., 2014). It is also described that activation of neurotensin receptor 1 has beneficial actions in a mouse model of Alzheimer's disease (Xiao et al., 2014). Considering that neurotensin activation is involved in cognition improvement, it seems reasonable to understand our findings of prenatal LPS reducing neurotensin levels and consequently impairing behavioral parameters of cognition (T-maze).

Moreover, the neurotensin reduction after prenatal LPS may be related to a dopaminergic disturbance. We previously reported that our rat model induces striatal dopaminergic disturbances in the offspring, such as decreased tyrosine hydroxylase expression as well as dopamine and metabolite level reduction (Kirsten et al., 2012, 2010b) as well as dopaminergic hypoactivity in the hypothalamus (Kirsten and Bernardi, 2017). Experimental evidence indicates intimate anatomical and biochemical relationships between the neurotensinergic and the dopaminergic systems (Binder et al., 2001; Rodríguez de Lores Arnaiz and Antonelli, 2016). Neurotensin and dopaminergic neurons are strongly co-located, and its receptors are expressed on both the dopaminergic neurons and on their postsynaptic targets (Muehlmann et al., 2018). Therefore, the neurotensin disturbance induced by prenatal LPS occurred possibly in response to the central dopaminergic impairment.

Future studies focusing on the central nervous system, especially for brain neurotensin levels and NTS1 and NTR3/sortilin receptors would be interesting for a better understanding of the mechanisms involved with ASD and neurotensin, including as a potential therapeutic target for autism.

In conclusion, exposure of rats to LPS during gestation (GD 9.5) induced cognitive inflexibility and elevated BDNF levels in juvenile male rat offspring, which are described as autistic-like symptoms. We also found reduced plasma neurotensin levels after prenatal LPS exposure. Daily postnatal pioglitazone treatment corrected the cognition impairments, as well as BDNF and neurotensin disturbances. Together with our previous studies (Kirsten et al., 2018), we suggested pioglitazone as a candidate for the treatment of autism, because it improved the responses of the three most typical autistic-like behaviors. BDNF and neurotensin also appeared to be related with autistic-like behaviors and should be considered for therapeutic purposes.

## MATERIALS AND METHODS

### Ethics statement

This study was performed in strict accordance with the recommendations in the Guide for the Care and Use of Laboratory Animals of the National Institutes of Health. The protocol was approved by the Committee on the Ethics of Animal Experiments of the School of Veterinary Medicine, University of São Paulo, Brazil (permit no. 2824/2012). All efforts were made to minimize the suffering, reduce the number of animals used and utilize alternatives to *in vivo* techniques when available. The experiments were performed in accordance with good laboratory practice protocols and quality assurance methods.

### Animals

Nineteen pregnant Wistar rats (*Rattus norvegicus*) of 15–17 weeks of age and weighing 220–275 g were used. The rats’ housing, nutritional conditions, determination of GD 0, pregnancy monitoring, weaning and litter sexing during the experiments were the same as previously described by our group (Kirsten et al., 2012, 2018).

### Prenatal treatments

LPS (from *Escherichia coli*; Sigma-Aldrich; serotype 0127: B8) was dissolved in sterile saline (50 µg/ml LPS in a 0.9% NaCl solution) and administered intraperitoneally (i.p.) to pregnant dams at a dose of 100 µg/kg on GD 9.5 (*n*=13). This GD of LPS exposure as well as the dose was chosen based on our previous studies (Kirsten et al., 2010a,b, 2013). Other dams received the vehicle (0.9% sterile saline, SAL) on GD 9.5 (*n*=6). Each dam (LPS and control) received 0.2 ml/100 g of each solution (LPS and vehicle solutions).

### Postnatal treatments and groups

The male offspring which were prenatally exposed to LPS (or SAL) also received pioglitazone (Sigma-Aldrich) or its vehicle dimethyl sulfoxide (DMSO, diluted to 1:10 in SAL) daily from PND 21 until 29. Rats received one of two pioglitazone doses: 0.25 and 1.0 mg/kg/day (i.p.) based on the pharmacokinetics of the drug (Actos, Abbott, Rio de Janeiro, Brazil), the autistic children's prescriptions (Boris et al., 2007) and rat studies (Park et al., 2007; Zhang et al., 2011; Alzamendi et al., 2012). The prepubertal period of the daily treatment was also based on an autistic children’s study (Boris et al., 2007) and the equivalent age between humans and rats (Quinn, 2005).

Four groups were investigated (*n*=8 rats per group): the SAL+DMSO group (control group), the LPS+DMSO group (LPS group), the LPS+PI0.25 group and the LPS+PI1.0 group. The experimental groups are described in Table 1.

**Table 1.**
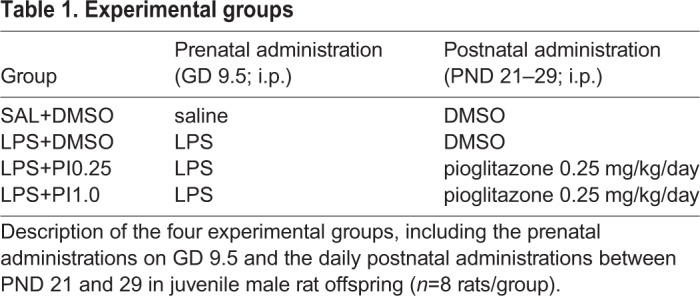
**Experimental groups**

### T-maze

One of the most typical symptoms of ASD is cognitive inflexibility (DSM-IV, 1994). To evaluate cognitive inflexibility, we used a T-maze; the T-maze specifications and behavioral protocol that were used during the experiments were the same as previously described by our group (Kirsten et al., 2015a; Cezar et al., 2018). The parameter analyzed was the alternation between the left and right arms after the five sessions. These data were transformed into scores: 0–4, i.e., from no alternations to four alternations.

### Plasma evaluations

On PND 36–40, the rats that were previously evaluated for their behavior were decapitated and plasma samples were obtained as previously described by our group (Moraes et al., 2017). BDNF (cat. no. G7610, Promega) analysis was performed using commercial enzyme-linked immunosorbent assay kits. Neuropeptides analysis, which included the analysis of beta-endorphin, neurotensin, oxytocin and substance P, was performed using Luminex/Magpix/Milliplex system (cat. no. RMNPMAG-83K, Millipore).

### Statistical analysis

Homogeneity and normality were verified using a Bartlett's test or *F* test. For the T-maze score analysis, a Kruskal–Wallis test was used, followed by a Dunn's test. One-way analysis of variance (ANOVA) followed by Fisher's LSD multiple comparison tests were used to compare the parametric data between the four groups. The results are expressed as the median (minimum and maximum) or the mean±s.e.m. In all cases, the results were considered as statistically signiﬁcant at *P*<0.05.

## References

[BIO041327C1] Al-Ayadhi L. Y. (2005). Pro-inflammatory cytokines in autistic children in central Saudi Arabia. *Neurosciences (Riyadh)* 10, 155-158. 22473229

[BIO041327C2] Alzamendi A., Giovambattista A., García M. E., Rebolledo O. R., Gagliardino J. J. and Spinedi E. (2012). Effect of pioglitazone on the fructose-induced abdominal adipose tissue dysfunction. *PPAR Res.* 2012, 259093 10.1155/2012/259093 23091482 PMC3469242

[BIO041327C3] Angelidou A., Francis K., Vasiadi M., Alysandratos K.-D., Zhang B., Theoharides A., Lykouras L., Sideri K., Kalogeromitros D. and Theoharides T. C. (2010). Neurotensin is increased in serum of young children with autistic disorder. *J. Neuroinflammation* 7, 48 10.1186/1742-2094-7-48 20731814 PMC2936302

[BIO041327C4] Ashdown H., Dumont Y., Ng M., Poole S., Boksa P. and Luheshi G. N. (2006). The role of cytokines in mediating effects of prenatal infection on the fetus: implications for schizophrenia. *Mol. Psychiatry* 11, 47-55. 10.1038/sj.mp.4001748 16189509

[BIO041327C5] Baio J., Wiggins L., Christensen D. L., Maenner M. J., Daniels J., Warren Z., Kurzius-Spencer M., Zahorodny W., Robinson C., Rosenberg C. et al. (2018). Prevalence of autism spectrum disorder among children aged 8 years - autism and developmental disabilities monitoring network, 11 sites, United States, 2014. *MMWR Surveill. Summ.* 67, 1-23. 10.15585/mmwr.ss6706a1 PMC5919599 29701730

[BIO041327C6] Bakker-Huvenaars M. J., Greven C. U., Herpers P., Wiegers E., Jansen A., Van Der Steen R., van Herwaarden A. E., Baanders A. N., Nijhof K. S., Scheepers F. et al. (2018). Saliva oxytocin, cortisol, and testosterone levels in adolescent boys with autism spectrum disorder, oppositional defiant disorder/conduct disorder and typically developing individuals. *Eur. Neuropsychopharmacol.* S0924-977X, 30280-30283. 10.1016/j.euroneuro.2018.07.097 30201120

[BIO041327C7] Berger J. and Wagner J. A. (2002). Physiological and therapeutic roles of peroxisome proliferator-activated receptors. *Diabetes Technol. Ther.* 4, 163-174. 10.1089/15209150260007381 12079620

[BIO041327C8] Binder D. K. and Scharfman H. E. (2004). Mini Review. *Growth Factors* 22, 123-131. 10.1080/08977190410001723308 15518235 PMC2504526

[BIO041327C9] Binder E. B., Kinkead B., Owens M. J. and Nemeroff C. B. (2001). Neurotensin and dopamine interactions. *Pharmacol. Rev.* 53, 453-486. 11734615

[BIO041327C10] Bongartz T., Coras B., Vogt T., Scholmerich J. and Muller-Ladner U. (2005). Treatment of active psoriatic arthritis with the PPARgamma ligand pioglitazone: an open-label pilot study. *Rheumatology (Oxf.)* 44, 126-129. 10.1093/rheumatology/keh423 15479756

[BIO041327C11] Boris M., Kaiser C. C., Goldblatt A., Elice M. W., Edelson S. M., Adams J. B. and Feinstein D. L. (2007). Effect of pioglitazone treatment on behavioral symptoms in autistic children. *J. Neuroinflammation* 4, 3 10.1186/1742-2094-4-3 17207275 PMC1781426

[BIO041327C12] Cai Z., Pan Z.-L., Pang Y., Evans O. B. and Rhodes P. G. (2000). Cytokine induction in fetal rat brains and brain injury in neonatal rats after maternal lipopolysaccharide administration. *Pediatr. Res.* 47, 64-72. 10.1203/00006450-200001000-00013 10625084

[BIO041327C13] Cezar L. C., Kirsten T. B., Da Fonseca C. C. N., De Lima A. P. N., Bernardi M. M. and Felicio L. F. (2018). Zinc as a therapy in a rat model of autism prenatally induced by valproic acid. *Prog. Neuropsychopharmacol. Biol. Psychiatry* 84, 173-180. 10.1016/j.pnpbp.2018.02.008 29481896

[BIO041327C14] Correia C. T., Coutinho A. M., Sequeira A. F., Sousa I. G., Lourenço Venda L., Almeida J. P., Abreu R. L., Lobo C., Miguel T. S., Conroy J. et al. (2010). Increased BDNF levels and NTRK2 gene association suggest a disruption of BDNF/TrkB signaling in autism. *Genes Brain Behav.* 9, 841-848. 10.1111/j.1601-183X.2010.00627.x 20662941

[BIO041327C15] Dobner P. R. (2005). Multitasking with neurotensin in the central nervous system. *Cell. Mol. Life Sci.* 62, 1946-1963. 10.1007/s00018-005-5128-x 16003489 PMC11139108

[BIO041327C16] DSM-IV. (1994). *Pervasive Developmental Disorders*, 4th edn., pp. 65-78. Washington, DC: American Psychiatric Association.

[BIO041327C17] Duval C., Chinetti G., Trottein F., Fruchart J.-C. and Staels B. (2002). The role of PPARs in atherosclerosis. *Trends Mol. Med.* 8, 422-430. 10.1016/S1471-4914(02)02385-7 12223313

[BIO041327C18] Feinstein D. L. (2003). Therapeutic potential of peroxisome proliferator-activated receptor agonists for neurological disease. *Diabetes Technol. Ther.* 5, 67-73. 10.1089/152091503763816481 12725709

[BIO041327C19] Garcia K. L. P., Yu G., Nicolini C., Michalski B., Garzon D. J., Chiu V. S., Tongiorgi E., Szatmari P. and Fahnestock M. (2012). Altered balance of proteolytic isoforms of pro-brain-derived neurotrophic factor in autism. *J. Neuropathol. Exp. Neurol.* 71, 289-297. 10.1097/NEN.0b013e31824b27e4 22437340 PMC3419254

[BIO041327C20] Ghaleiha A., Rasa S. M., Nikoo M., Farokhnia M., Mohammadi M.-R. and Akhondzadeh S. (2015). A pilot double-blind placebo-controlled trial of pioglitazone as adjunctive treatment to risperidone: effects on aberrant behavior in children with autism. *Psychiatry Res.* 229, 181-187. 10.1016/j.psychres.2015.07.043 26208985

[BIO041327C21] Ghanizadeh A. (2010). Targeting neurotensin as a potential novel approach for the treatment of autism. *J. Neuroinflammation* 7, 58 10.1186/1742-2094-7-58 20920308 PMC2958902

[BIO041327C22] Gillberg C. (1992). Plasma beta-endorphin concentrations in autism. *J. Autism Dev. Disord.* 22, 131-133. 10.1007/BF01046409 1592763

[BIO041327C23] Gillies P. S. and Dunn C. J. (2000). Pioglitazone. *Drugs* 60, 333-343; discussion 44-45 10.2165/00003495-200060020-00009 10983737

[BIO041327C24] Hanefeld M. and Belcher G. (2001). Safety profile of pioglitazone. *Int. J. Clin. Pract. Suppl.* 121, 27-31. 11594241

[BIO041327C25] Herbert M. R. (2010). Contributions of the environment and environmentally vulnerable physiology to autism spectrum disorders. *Curr. Opin. Neurol.* 23, 103-110. 10.1097/WCO.0b013e328336a01f 20087183

[BIO041327C26] Johnson N. L., Giarelli E., Lewis C. and Rice C. E. (2013). Genomics and autism spectrum disorder. *J. Nurs. Scholarsh.* 45, 69-78. 10.1111/j.1547-5069.2012.01483.x 23368711 PMC4450805

[BIO041327C27] Jyonouchi H., Sun S. and Le H. (2001). Proinflammatory and regulatory cytokine production associated with innate and adaptive immune responses in children with autism spectrum disorders and developmental regression. *J. Neuroimmunol.* 120, 170-179. 10.1016/S0165-5728(01)00421-0 11694332

[BIO041327C28] Katayama K., Wada K., Nakajima A., Mizuguchi H., Hayakawa T., Nakagawa S., Kadowaki T., Nagai R., Kamisaki Y., Blumberg R. S. et al. (2003). A novel PPAR gamma gene therapy to control inflammation associated with inflammatory bowel disease in a murine model. *Gastroenterology* 124, 1315-1324. 10.1016/S0016-5085(03)00262-2 12730872

[BIO041327C29] Keiser A. A., Matazel K. S., Esser M. K., Feifel D. and Prus A. J. (2014). Systemic administration of the neurotensin NTS(1)-receptor agonist PD149163 improves performance on a memory task in naturally deficient male brown Norway rats. *Exp. Clin. Psychopharmacol.* 22, 541-547. 10.1037/a0037912 25222546 PMC4250335

[BIO041327C30] Kirsten T. B. and Bernardi M. M. (2017). Prenatal lipopolysaccharide induces hypothalamic dopaminergic hypoactivity and autistic-like behaviors: repetitive self-grooming and stereotypies. *Behav. Brain Res.* 331, 25-29. 10.1016/j.bbr.2017.05.013 28526515

[BIO041327C31] Kirsten T. B., Taricano M., Maiorka P. C., Palermo-Neto J. and Bernardi M. M. (2010a). Prenatal lipopolysaccharide reduces social behavior in male offspring. *Neuroimmunomodulation* 17, 240-251. 10.1159/000290040 20203530

[BIO041327C32] Kirsten T. B., Taricano M., Flório J. C., Palermo-Neto J. and Bernardi M. M. (2010b). Prenatal lipopolysaccharide reduces motor activity after an immune challenge in adult male offspring. *Behav. Brain Res.* 211, 77-82. 10.1016/j.bbr.2010.03.009 20226214

[BIO041327C33] Kirsten T. B., Chaves-Kirsten G. P., Chaible L. M., Silva A. C., Martins D. O., Britto L. R. G., Dagli M. L. Z., Torrão A. S., Palermo-Neto J. and Bernardi M. M. (2012). Hypoactivity of the central dopaminergic system and autistic-like behavior induced by a single early prenatal exposure to lipopolysaccharide. *J. Neurosci. Res.* 90, 1903-1912. 10.1002/jnr.23089 22714803

[BIO041327C34] Kirsten T. B., Lippi L. L., Bevilacqua E. and Bernardi M. M. (2013). LPS exposure increases maternal corticosterone levels, causes placental injury and increases IL-1β levels in adult rat offspring: relevance to autism. *PLoS ONE* 8, e82244 10.1371/journal.pone.0082244 24312647 PMC3846733

[BIO041327C35] Kirsten T. B., Chaves-Kirsten G. P., Bernardes S., Scavone C., Sarkis J. E., Bernardi M. M. and Felicio L. F. (2015a). Lipopolysaccharide exposure induces maternal hypozincemia, and prenatal zinc treatment prevents autistic-like behaviors and disturbances in the striatal dopaminergic and mTOR systems of offspring. *PLoS ONE* 10, e0134565 10.1371/journal.pone.0134565 26218250 PMC4517817

[BIO041327C36] Kirsten T. B., Queiroz-Hazarbassanov N., Bernardi M. M. and Felicio L. F. (2015b). Prenatal zinc prevents communication impairments and BDNF disturbance in a rat model of autism induced by prenatal lipopolysaccharide exposure. *Life Sci.* 130, 12-17. 10.1016/j.lfs.2015.02.027 25817235

[BIO041327C37] Kirsten T. B., Casarin R. C., Bernardi M. M. and Felicio L. F. (2018). Pioglitazone abolishes autistic-like behaviors via the IL-6 pathway. *PLoS ONE* 13, e0197060 10.1371/journal.pone.0197060 29791472 PMC5965820

[BIO041327C38] Landreth G. (2006). PPARgamma agonists as new therapeutic agents for the treatment of Alzheimer's disease. *Exp. Neurol.* 199, 245-248. 10.1016/j.expneurol.2006.04.006 16733054

[BIO041327C39] Landrigan P. J. (2010). What causes autism? Exploring the environmental contribution. *Curr. Opin. Pediatr.* 22, 219-225. 10.1097/MOP.0b013e328336eb9a 20087185

[BIO041327C40] Larsson H. J., Eaton W. W., Madsen K. M., Vestergaard M., Olesen A. V., Agerbo E., Schendel D., Thorsen P. and Mortensen P. B. (2005). Risk factors for autism: perinatal factors, parental psychiatric history, and socioeconomic status. *Am. J. Epidemiol.* 161, 916-928. 10.1093/aje/kwi123 15870155

[BIO041327C41] Li X., Chauhan A., Sheikh A. M., Patil S., Chauhan V., Li X.-M., Ji L., Brown T. and Malik M. (2009). Elevated immune response in the brain of autistic patients. *J. Neuroimmunol.* 207, 111-116. 10.1016/j.jneuroim.2008.12.002 19157572 PMC2770268

[BIO041327C42] Marui T., Funatogawa I., Koishi S., Yamamoto K., Matsumoto H., Hashimoto O., Nanba E., Nishida H., Sugiyama T., Kasai K. et al. (2007). Tachykinin 1 (TAC1) gene SNPs and haplotypes with autism: a case-control study. *Brain Dev.* 29, 510-513. 10.1016/j.braindev.2007.01.010 17376622

[BIO041327C43] Mcpheeters M. L., Warren Z., Sathe N., Bruzek J. L., Krishnaswami S., Jerome R. N. and Veenstra-Vanderweele J. (2011). A systematic review of medical treatments for children with autism spectrum disorders. *Pediatrics* 127, e1312-e1321. 10.1542/peds.2011-0427 21464191

[BIO041327C44] Moraes M. M. T., Galvão M. C., Cabral D., Coelho C. P., Queiroz-Hazarbassanov N., Martins M. F. M., Bondan E. F., Bernardi M. M. and Kirsten T. B. (2017). Propentofylline prevents sickness behavior and depressive-like behavior induced by lipopolysaccharide in rats via neuroinflammatory pathway. *PLoS ONE* 12, e0169446 10.1371/journal.pone.0169446 28056040 PMC5215944

[BIO041327C45] Muehlmann A. M., Wolfman S. L. and Devine D. P. (2018). The role of neurotensin in vulnerability for self-injurious behaviour: studies in a rodent model. *J. Intellect. Disabil. Res.* 62, 997-1007. 10.1111/jir.12519 30033601

[BIO041327C46] Nelson K. B., Grether J. K., Croen L. A., Dambrosia J. M., Dickens B. F., Jelliffe L. L., Hansen R. L. and Phillips T. M. (2001). Neuropeptides and neurotrophins in neonatal blood of children with autism or mental retardation. *Ann. Neurol.* 49, 597-606. 10.1002/ana.1024 11357950

[BIO041327C47] Park S.-W., Yi J.-H., Miranpuri G., Satriotomo I., Bowen K., Resnick D. K. and Vemuganti R. (2007). Thiazolidinedione class of peroxisome proliferator-activated receptor gamma agonists prevents neuronal damage, motor dysfunction, myelin loss, neuropathic pain, and inflammation after spinal cord injury in adult rats. *J. Pharmacol. Exp. Ther.* 320, 1002-1012. 10.1124/jpet.106.113472 17167171

[BIO041327C48] Patel A. B., Tsilioni I., Leeman S. E. and Theoharides T. C. (2016). Neurotensin stimulates sortilin and mTOR in human microglia inhibitable by methoxyluteolin, a potential therapeutic target for autism. *Proc. Natl. Acad. Sci. USA* 113, E7049-E7E58. 10.1073/pnas.1604992113 27663735 PMC5111711

[BIO041327C49] Posar A. and Visconti P. (2017). Autism in 2016: the need for answers. *J. Pediatr.* 93, 111-119. 10.1016/j.jped.2016.09.002 27837654

[BIO041327C50] Prakash A. and Kumar A. (2014). Role of nuclear receptor on regulation of BDNF and neuroinflammation in hippocampus of beta-amyloid animal model of Alzheimer's disease. *Neurotox. Res.* 25, 335-347. 10.1007/s12640-013-9437-9 24277156

[BIO041327C51] Quattrocki E. and Friston K. (2014). Autism, oxytocin and interoception. *Neurosci. Biobehav. Rev.* 47, 410-430. 10.1016/j.neubiorev.2014.09.012 25277283 PMC4726659

[BIO041327C52] Quinn R. (2005). Comparing rat's to human's age: how old is my rat in people years? *Nutrition* 21, 775-777. 10.1016/j.nut.2005.04.002 15925305

[BIO041327C53] Ricci S., Businaro R., Ippoliti F., Lo Vasco V. R., Massoni F., Onofri E., Troili G. M., Pontecorvi V., Morelli M., Rapp Ricciardi M. et al. (2013). Altered cytokine and BDNF levels in autism spectrum disorder. *Neurotox. Res.* 24, 491-501. 10.1007/s12640-013-9393-4 23604965

[BIO041327C54] Rodríguez De Lores Arnaiz G. and Antonelli M. C. (2016). In search of concomitant alterations of dopaminergic and neurotensinergic systems in stress conditions. *Neurochem. Res.* 41, 423-430. 10.1007/s11064-016-1849-5 26869038

[BIO041327C55] Theoharides T. C., Kempuraj D. and Redwood L. (2009). Autism: an emerging ‘neuroimmune disorder’ in search of therapy. *Expert Opin Pharmacother.* 10, 2127-2143. 10.1517/14656560903107789 19640207

[BIO041327C56] Tordjman S., Anderson G. M., Botbol M., Brailly-Tabard S., Perez-Diaz F., Graignic R., Carlier M., Schmit G., Rolland A.-C., Bonnot O. et al. (2009). Pain reactivity and plasma beta-endorphin in children and adolescents with autistic disorder. *PLoS ONE* 4, e5289 10.1371/journal.pone.0005289 19707566 PMC2728512

[BIO041327C57] Tordjman S., Somogyi E., Coulon N., Kermarrec S., Cohen D., Bronsard G., Bonnot O., Weismann-Arcache C., Botbol M., Lauth B. et al. (2014). Gene x Environment interactions in autism spectrum disorders: role of epigenetic mechanisms. *Front. Psychiatry* 5, 53 10.3389/fpsyt.2014.00053 25136320 PMC4120683

[BIO041327C58] Tsilioni I., Dodman N., Petra A. I., Taliou A., Francis K., Moon-Fanelli A., Shuster L. and Theoharides T. C. (2014). Elevated serum neurotensin and CRH levels in children with autistic spectrum disorders and tail-chasing Bull Terriers with a phenotype similar to autism. *Transl. Psychiatry* 4, e466 10.1038/tp.2014.106 25313509 PMC5190146

[BIO041327C59] Urakubo A., Jarskog L. F., Lieberman J. A. and Gilmore J. H. (2001). Prenatal exposure to maternal infection alters cytokine expression in the placenta, amniotic fluid, and fetal brain. *Schizophr. Res.* 47, 27-36. 10.1016/S0920-9964(00)00032-3 11163542

[BIO041327C60] Vincent J.-P., Mazella J. and Kitabgi P. (1999). Neurotensin and neurotensin receptors. *Trends Pharmacol. Sci.* 20, 302-309. 10.1016/S0165-6147(99)01357-7 10390649

[BIO041327C61] Weizman R., Gil-Ad I., Dick J., Tyano S., Szekely G. A. and Laron Z. (1988). Low plasma immunoreactive beta-endorphin levels in autism. *J. Am. Acad. Child Adolesc. Psychiatry* 27, 430-433. 10.1097/00004583-198807000-00009 2972691

[BIO041327C62] Wink L. K., Plawecki M. H., Erickson C. A., Stigler K. A. and Mcdougle C. J. (2010). Emerging drugs for the treatment of symptoms associated with autism spectrum disorders. *Expert Opin Emerg. Drugs* 15, 481-494. 10.1517/14728214.2010.487860 20470188 PMC2923687

[BIO041327C63] Xiao Z., Cilz N. I., Kurada L., Hu B., Yang C., Wada E., Combs C. K., Porter J. E., Lesage F. and Lei S. (2014). Activation of neurotensin receptor 1 facilitates neuronal excitability and spatial learning and memory in the entorhinal cortex: beneficial actions in an Alzheimer's disease model. *J. Neurosci.* 34, 7027-7042. 10.1523/JNEUROSCI.0408-14.2014 24828655 PMC4019809

[BIO041327C64] Yamanaka M., Itakura Y., Tsuchida A., Nakagawa T., Noguchi H. and Taiji M. (2007). Comparison of the antidiabetic effects of brain-derived neurotrophic factor and thiazolidinediones in obese diabetic mice. *Diabetes Obes. Metab.* 9, 879-888. 10.1111/j.1463-1326.2006.00675.x 17924870

[BIO041327C65] Yang S., Dong X., Guo X., Han Y., Song H., Gao L., Dai W., Su Y. and Zhang X. (2017). Serum oxytocin levels and an oxytocin receptor gene polymorphism (rs2254298) indicate social deficits in children and adolescents with autism spectrum disorders. *Front. Neurosci.* 11, 221 10.3389/fnins.2017.00221 28484366 PMC5399030

[BIO041327C66] Zhang H.-L., Xu M., Wei C., Qin A.-P., Liu C.-F., Hong L.-Z., Zhao X.-Y., Liu J. and Qin Z.-H. (2011). Neuroprotective effects of pioglitazone in a rat model of permanent focal cerebral ischemia are associated with peroxisome proliferator-activated receptor gamma-mediated suppression of nuclear factor-kappaB signaling pathway. *Neuroscience* 176, 381-395. 10.1016/j.neuroscience.2010.12.029 21185913

[BIO041327C67] Zhang H.-F., Dai Y.-C., Wu J., Jia M.-X., Zhang J.-S., Shou X.-J., Han S.-P., Zhang R. and Han J.-S. (2016). Plasma oxytocin and arginine-vasopressin levels in children with autism spectrum disorder in china: associations with symptoms. *Neurosci. Bull.* 32, 423-432. 10.1007/s12264-016-0046-5 27342432 PMC5563759

